# YopH inhibits early pro-inflammatory cytokine responses during plague pneumonia

**DOI:** 10.1186/1471-2172-11-29

**Published:** 2010-06-16

**Authors:** Angelene M Cantwell, Sarah S Bubeck, Peter H Dube

**Affiliations:** 1 Department of Microbiology and Immunology, The University of Texas Health Science Center at San Antonio, 7703 Floyd Curl Dr, San Antonio, TX 78229, USA; 2 Department of Biology, University of Texas at San Antonio, One UTSA circle, San Antonio, TX 78249, USA

## Abstract

**Background:**

*Yersinia pestis *is the causative agent of pneumonic plague; recently, we and others reported that during the first 24-36 hours after pulmonary infection with *Y. pestis *pro-inflammatory cytokine expression is undetectable in lung tissues.

**Results:**

Here, we report that, intranasal infection of mice with CO92 *delta yopH *mutant results in an early pro-inflammatory response in the lungs characterized by an increase in the pro-inflammatory cytokines Tumor Necrosis Factor-alpha and Interleukin one-beta 24 hours post-infection. CO92 *delta yopH *colonizes the lung but does not disseminate to the liver or spleen and is cleared from the host within 72 hours post-infection. This is different from what is observed in a wild-type CO92 infection, where pro-inflammatory cytokine expression and immune cell infiltration into the lungs is not detectable until 36-48 h post-infection. CO92 rapidly disseminates to the liver and spleen resulting in high bacterial burdens in these tissues ultimately cumulating in death 72-94 h post-infection. Mice deficient in TNF-alpha are more susceptible to CO92 *delta yopH *infection with 40% of the mice succumbing to infection.

**Conclusions:**

Altogether, our results suggest that YopH can inhibit an early pro-inflammatory response in the lungs of mice and that this is an important step in the pathogenesis of infection.

## Background

*Yersinia pestis *is a Gram-negative bacterium with a zoonotic life cycle that occasionally results in human infections leading to plague [1]. Plague manifests in two major forms, the most common being bubonic plague. Bubonic plague is transmitted to humans through the bite of an infected flea, resulting in intradermal inoculation of the bacterium, which progresses to form the characteristic lymphadenitis (bubos) of plague [1]. The less common but contagious form of the disease is pneumonic plague, which is a severe pneumonia resulting either from inhalation of infectious respiratory droplets, or secondary to bubonic or septicemic plague. Pneumonic plague is highly aggressive and if untreated, is able to kill the host within 2-4 days post-exposure, with mortality rates that approach 100% [1-3].

We and others have begun to analyze the immune response to primary pneumonic plague using wild-type strains of *Y. pestis *in an effort to understand the interactions of *Y. pestis *with its host [4,5]early during the development of disease. The rapid progression of the disease and associated mortality suggests that subversion of innate immunity plays a key role in disease development [4]. Our previous studies indicate a delay in the host inflammatory response to infection, resulting in an opportunity for the bacterium to replicate to high numbers and potentially overwhelm the host immune system, rapidly resulting in death [4]. Modulation of host-immune responses is a common pathogenic mechanism among the virulent species of *Yersinia *and much work has been done to understand the molecular mechanisms underlying the virulence factors involved in this process [6-8].

The 70 Kb virulence plasmid (pCD-1), which is essential for virulence, contains all of the machinery and effector proteins for a type-three secretion system (TTSS) [6,8]. The secreted effector proteins are also known as the *Yersinia *outer proteins (Yops) and are transported from the bacterial cytosol through the TTSS into the cytoplasm of a host cell to facilitate infection. All six of the effector Yops have been studied, and at least one function has been assigned to each based on in vitro studies. Several are involved in manipulating the host cytoskeleton (YopE, YopH, YopT, YopO); these yops interfere with the Rho family of GTPases and other host proteins involved in the regulation of the cytoskeleton, and are therefore important in the modulation of phagocytosis [9,10]. Others are involved in the tempering of the host immune response, in particular, YopH and YopJ, and to a lesser extent, YopE and YopM have been shown to impact inflammatory cytokine expression in vitro and in vivo respectively [8,11].

YopH is a protein tyrosine phosphatase, which is known to interact with p130cas and FAK to impair invasion of epithelial cells by *Yersinia pseudotuberculosis *[12,13]. YopH blocks cytoskeletal rearrangement upon injection into the host cell, contributing to the decreased ability of macrophages and other immune cells to phagocytose the bacteria [14]. Distinct from its ability to interact with the cytoskeleton, YopH is also known to impact host cell signaling by inactivation of the PI3K pathway through unknown mechanisms [15]. The PI3K pathway plays an important role in the macrophage response to infection including generation of the oxidative burst and nitric oxide production, phagosome formation, and has a role in a negative regulation of IL-12 production, to help keep the inflammatory response in check [16].

Numerous studies have determined that mutations in YopH severely impact the virulence of the yersiniae [17-19]. Recently we reported that CO92*ΔyopH *is severely attenuated in both an intranasal and subcutaneous models of *Y. pesti*s infection, which makes CO92*ΔyopH *a very good live-attenuated vaccine strain [18]. The ability of CO92*ΔyopH *to provide significant protection against virulent challenge suggests that a robust protective immune response is generated during primary infection with this strain. However, the role of YopH in the pathogenesis of plague pneumonia or its impact on the immune response in the lung has not been examined. In this study we test the impact of the CO92*ΔyopH *mutant on both the virulence and inflammatory response using a mouse model of primary pneumonic plague.

## Results

### *Y. pestis *CO92*ΔyopH *is severely attenuated in an intranasal model of primary pneumonic plague

**Figure 1:**
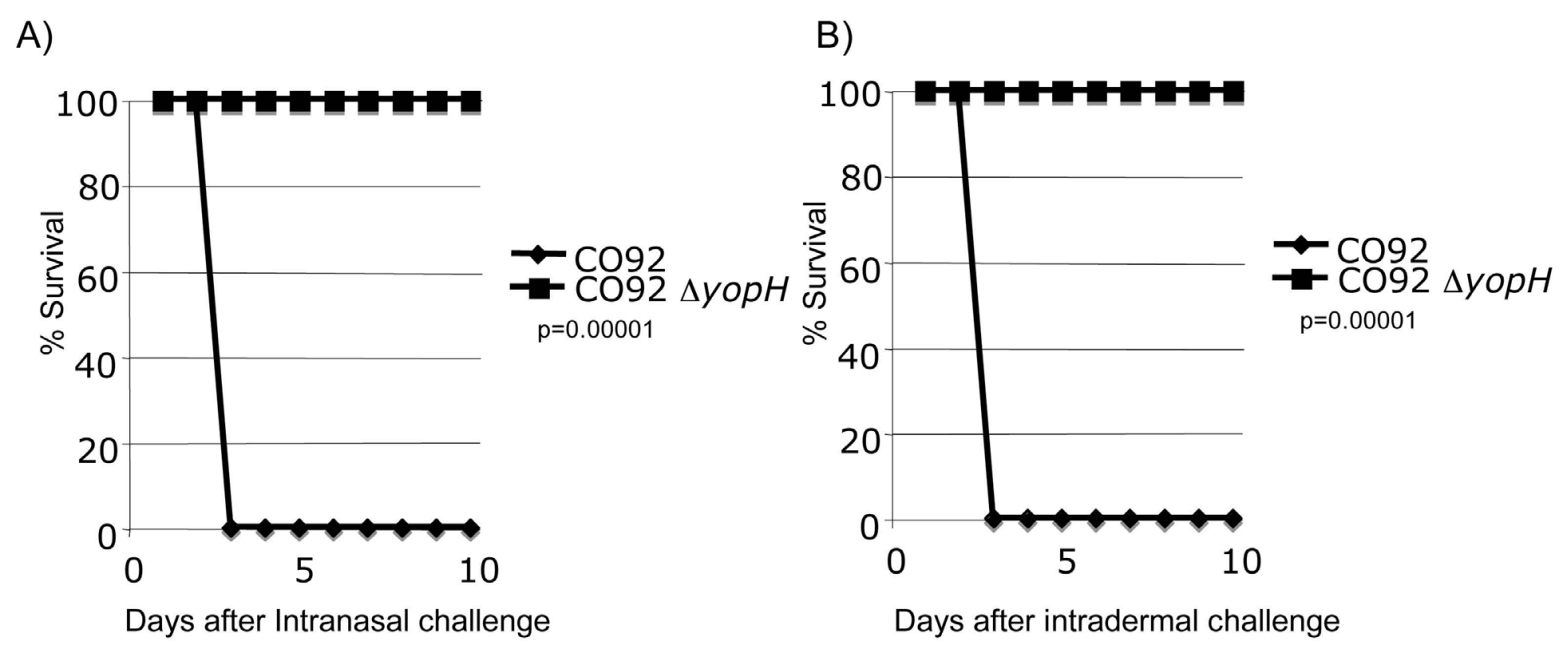
**CO92Δ*yopH *is attenuated by several routes of infection**. **A) **6-8 week old CD-1 mice were infected IN with 2 × 10^4 ^CFU of CO92 or 1 × 10^7 ^CFU CO92Δ*yopH*. The mice were monitored for survival over a 10-day period. **B) **Mice were infected by intradermal injection with 2 × 10^4 ^CFU of CO92 or 1 × 10^7 ^CFU CO92Δ*yopH *and then monitored for survival. Data was analyzed by log-rank analysis and determined to be significant in both cases p = 0.00001. Data is representative of three independent experiments with 10/mice per experimental group.

The survival of CD1 mice after IN infection with CO92 or CO92*ΔyopH *was determined and the survival curves analyzed by log-rank analysis. Mice infected IN with ~10^7 ^CFU CO92*ΔyopH *appeared normal at all time points post infection and showed no outward signs of disease whereas animals infected with ~10^4 ^CFU CO92 were severely debilitated two days post-infection and succumbed to disease by day 4 post-infection (Figure 1). Altogether, these data revealed that mice were able to survive both intranasal and intradermal infection with CO92*ΔyopH *at doses of ~10^7 ^CFU (Figure 1). Our results indicate a significant difference in survival (p = 0.00001) when CO92 is compared to CO92*ΔyopH *and that at the highest dose tested (~10^7^) CO92*ΔyopH *is avirulent in CD1 mice.

### Attenuation of CO92*ΔyopH *is due to the loss of *YopH *and *YopH *enzymatic activity

**Figure 2:**
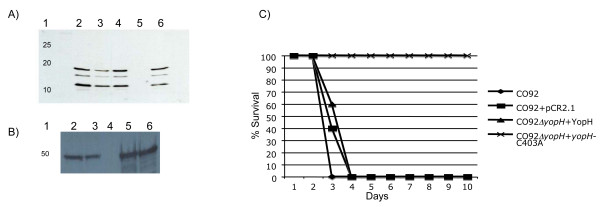
**YopH phosphatase activity is required for *Y. pestis *virulence**. A) Complementation of the *yopH *mutation leads to similar levels of YopH expression in vitro. Various strains of *Y. pestis *were grown under type-three secretion system inducing conditions and then the supernatant was subjected SDS-PAGE followed by immuno-blotting with rabbit-polyclonal anti-YopH antibodies. Immunoreactive protein bands were detected by enhanced chemiluminescence. Lanes: 1) molecular weight markers, 2&3) CO92, 4) CO92+pCR2.1, 5) CO92Δ*yopH *6) CO92Δ*yopH*+ pAMC-1 (YopH). Note multiple bands are due to Pla mediated proteolysis. **B) **Whole cell extracts of cells grown under type-three secretion system inducing conditions. Lanes: 1) molecular weight markers, 2) CO92, 4) CO92+pCR2.1, 5) CO92Δ*yopH *6) CO92Δ*yopH*+ pAMC-1 (YopH). **C) **Complementation of the *yopH *mutation restores virulence in vivo. CD-1 mice were infected IN with 2 × 10^4 ^CFU of various *Y. pestis *strains. Mice infected IN with CO92 (υ) or CO92+pCR2.1 (() succumbed rapidly to infection. Mice infected IN with CO92ΔyopH+pAMC-1 (σ), YopH, died with similar kinetics. However, mice infected IN with CO92ΔyopH+pAMC-2 ((), yopH-C403A, survived infection. Data is representative of two independent experiments with 10 mice/group.

We tested the ability of the CO92*ΔyopH *mutant complemented with a wild type copy of the yopH gene to secrete YopH in vitro. As shown in Figure 2A when the TTSS is induced in vitro, the CO92*ΔyopH *mutant complemented with yopH secretes similar levels of YopH into the culture supernatant as CO92. Due to the YopH cleavage products in secreted protein preparations, we also evaluated the levels of YopH in whole cell extracts after induction. Similar levels of YopH were detected in the whole cell extracts and were consistent with the secreted protein profile (Figure 2B). To test if the attenuation phenotype of CO92*ΔyopH *is linked to the *yopH *mutation, mice were infected with the CO92*ΔyopH *mutant carrying the pAMC-1 complementing plasmid. Complementation of YopH expressed from the pAMC-1 plasmid restores virulence to the levels observed with CO92 carrying the empty pCR2.1 plasmid (Figure 2C). However, virulence is lost when the CO92*ΔyopH *mutant is complemented with the YopH-C403A gene expressed from the pAMC-2 plasmid (Figure 2C). The C403A mutation abolishes the YopH tyrosine phophatase activity, strongly suggesting that the attenuation of the CO92*ΔyopH *mutant is due to loss of the YopH phosphatase activity and these data are consistent with previous studies of other *Yersinia *species and attenuated *Y. pestis *strains which determined that *yopH *mutants are highly attenuated [11,17,18,20,21].

### *Y. pestis *CO92*ΔyopH *is able to persist in the lungs but does not spread systemically

**Figure 3:**
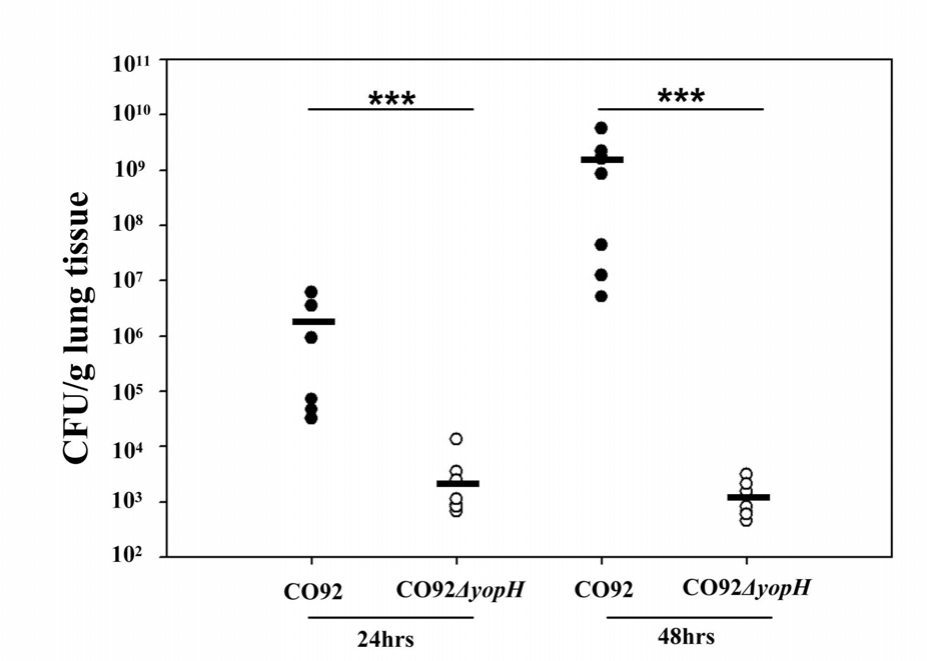
**Bacterial burdens in the Lungs**. 6-8 week old female CD1 mice were infected IN with 2×10^4^-1×10^5 ^CFU of CO92 or CO92*ΔyopH *respectively. Mice were sacrificed at 24 h and 48 h post-infection and the lungs were weighed, crushed, and plated for CFU/g tissue. There is a significant difference in bacterial colonization of the lungs between wild type CO92 and CO92*ΔyopH *at both 24 h (*** p = 0.0002) and 48 h (***p = 0.0002) post-infection. These data are representative of two independent experiments with at least 6 mice per group. *** p = 0.0002, Mann-Whitney U test, CO92 infected lungs vs. CO92*ΔyopH *infected lungs at 24 h or 48 h post-infection, bar represents mean value.

The severe attenuation of *Y. pestis *CO92*ΔyopH *could be due to an inability to colonize the lung, disseminate, or replicate in the mouse. To further investigate the pathogenesis of CO92*ΔyopH*, we determined the changes in bacterial burdens over time. Mice were infected IN with 2×10^4 ^or 1×10^5 ^CFU *Y. pestis *CO92 or CO92*ΔyopH *respectively and sacrificed at the indicated times. Lungs, livers, and spleens were processed as described in the methods and dilutions plated to determine the CFU-bacteria/g tissue at each time point post-infection (24 h, 48 h, 72 h, 96 h). All of the mice infected with wild-type CO92 were dead by 72 hours post-infection making collection of data at 72 h and beyond impossible for this group. Our results suggest that CO92*ΔyopH *is able to persist in the lungs through 48 h post-infection (Figure 3 and data not shown). Bacterial burdens in the lungs of mice infected with CO92 were significantly higher when compared to CO92*ΔyopH *at both 24 h and 48 h post-infection (p = 0.0002 for both time points, Mann-Whitney U test) (Figure 3). These data indicate that the CO92*ΔyopH *mutant was unable to replicate at the site of infection. In addition, we were unable to detect dissemination of the CO92*ΔyopH *bacterium to the spleen or liver at any time post-infection, while wild-type CO92 was present in the livers and spleens in large numbers at 48 h post-infection consistent with what we have reported previously [4].

### Intranasal infection with CO92*ΔyopH *induces TNF-α and IL-1β early during infection

**Figure 4:**
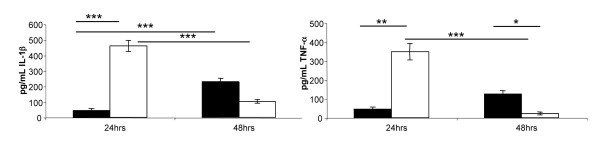
**Pro-inflammatory cytokines in the BALF of infected mice**. 6-8 week old female CD1 mice were infected with 2×10^4 ^CFU of either CO92 or CO92*ΔyopH*. Mice were sacrificed at 24 h and 48 h post-infection and bronchoalveolar lavage was performed on their lungs. BALF was collected, filtered, and assayed by ELISA for the presence of TNF-α and IL-1β. Levels of TNF-α (**A**) and IL-1β (**B**) are significantly higher at 24 h post-infection in mice infected with *Y. pestis *CO92Δ*yopH *as compared to mice infected with wild-type CO92. ■ = mice infected with wild-type *Y. pestis *CO92, □ = mice infected with *Y. pestis *CO92Δ*yopH*, *** p < 0.001, ** p < 0.01, * p < 0.05, non-parametric ANOVA, these data represent 8-12 samples per data point assayed in duplicate, and are from two independent experiments.

In mouse models of primary pneumonic plague, mice infected with CO92 do not mount an inflammatory cytokine response until 36 h to 48 h post-infection [4,5]. We hypothesized that the attenuation of CO92*ΔyopH *could be partially due to a strong inflammatory response to the presence of the bacterium. Further, due to the delayed arrival of neutrophils during plague pneumonia, we suspected that TNF-α and IL-1β might be involved [4,5]. To test this hypothesis, we measured the concentrations of TNF-α and IL-1β in the bronchiolar alveolar lavage fluid (BALF) after IN infection with CO92 or CO92*ΔyopH*. Mice were infected with 2×10^4 ^CFU of either *Y. pestis *CO92 or CO92*ΔyopH *and sacrificed 24 or 48 hours post-infection. BAL was performed on these mice, and then ELISA analyzed the BALF for the concentration of TNF-α and IL-1β. Mice infected with CO92*ΔyopH *elicited a robust pro-inflammatory cytokine response at 24 h post-infection, characterized by 349 ± 43 pg/ml of TNF-α and 461 ± 35 pg/ml of IL-1β in the BALF of these mice 24 h post-infection (p = 0.001 in comparison to CO92). By 48 h post-infection the levels of both cytokines had decreased but remained significant with concentrations of TNF-α = 45 ± 12 pg/ml, and IL-1β = 104 ± 12 pg/ml (Figures 4A & 4B). BAL from mice infected for 24 hours with wild-type CO92 had TNF-α and IL-1β levels approximately equal to the levels in the BALF from uninfected mice. However, at 48 h post-infection, both cytokines were detected in the BALF of these mice with concentrations of TNF-α = 125 ± 19 pg/ml, and IL-1β = 231 ± 21 pg/ml (Figures 4A &4B). The timing of cytokine expression and the levels of cytokine induced during a CO92 are consistent with what we have observed previously [[4], and data not shown]. Altogether, these data suggest that *YopH *is involved in delaying or inhibiting the expression of TNF-α and IL-1β during plague pneumonia and may be involved in the overall delayed inflammatory response to this infection.

### Mice lacking proinflammatory cytokines are more sensitive to CO92*ΔyopH *infection

**Figure 5:**
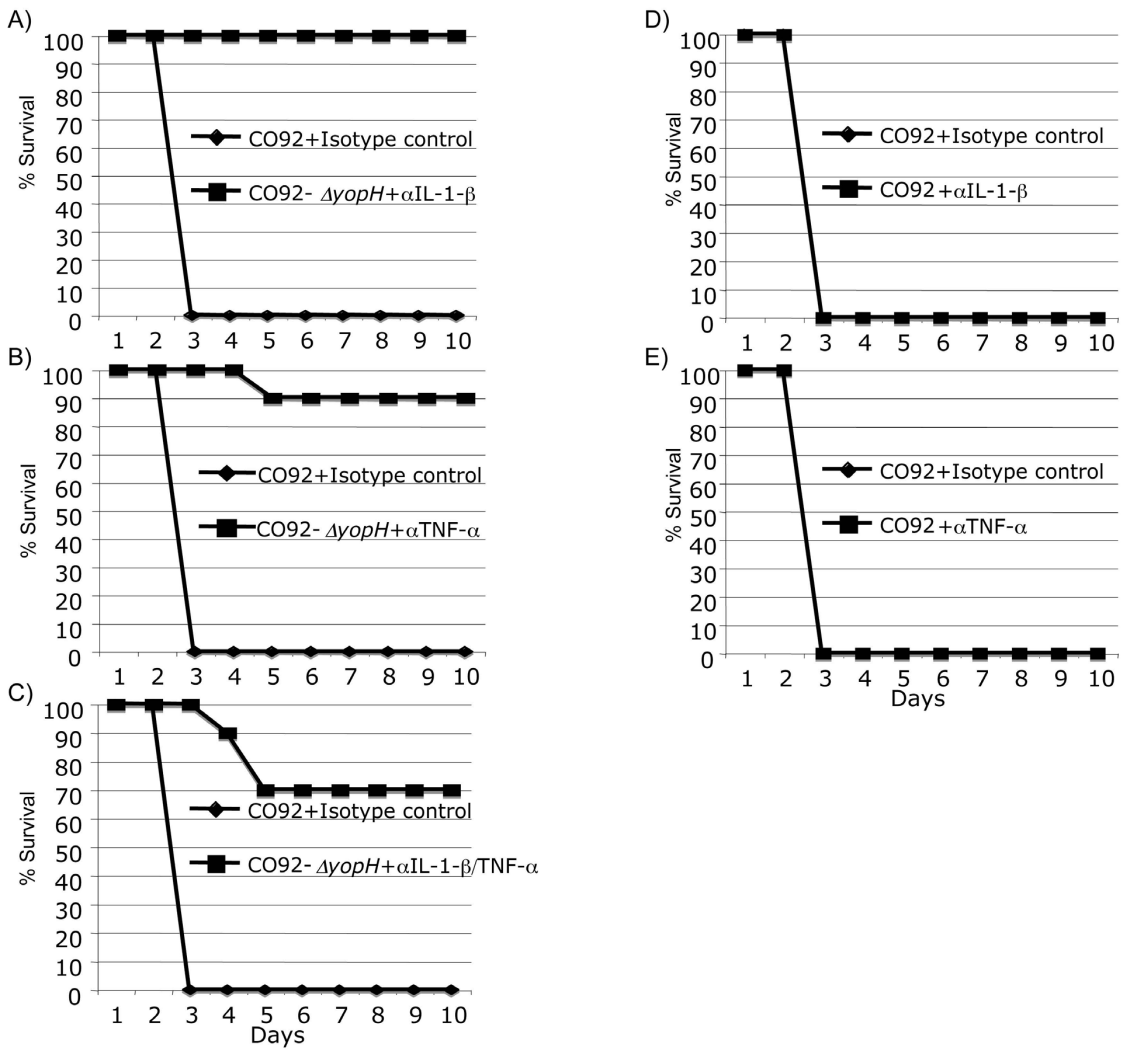
**Antibody mediated depletion of TNF-α or IL-1β enhances virulence of CO92Δ*yopH***. CD-1 mice were treated with cytokine depleting antibodies or isotype control antibodies 1 day prior to infection and then every third day for the course of the experiment. Animals were then infected IN with 2 X10^4 ^CFU of CO92 or CO92Δ*yopH *and monitored for survival for 10 days. **A) **Depletion of IL-1β has no effect. **B) **Depletion of TNF-α causes 10% mortality. **C) **Depletion of both TNF-α and IL-1β has a synergistic effect leading to 30% mortality. **D) **Depletion of IL-1β during CO92 infection has no effect. **E) **Depletion of TNF-α during CO92 infection has no effect. Data is combined from two independent experiments with 10 mice per group.

If TNF-α and IL-1β were critical host-components leading to the attenuation observed in mice infected with CO92*ΔyopH*, then it would not be unreasonable to predict that mice deficient in these molecules would be more sensitive to infection with CO92*ΔyopH*. To test this hypothesis, we used antibody mediated cytokine depletion followed by IN challenge with CO92*ΔyopH *or CO92. Briefly, mice were treated with monoclonal antibodies to TNF-α, IL-1β, TNF-α+IL-1β, or the appropriate control IgG one day prior to infection and then every third day for the course of the infection as we have described previously [22]. Mice treated with IL-1β depleting antibodies were not significantly more sensitive to CO92*ΔyopH *infection than the control mice (Figure 5A) and only 10% of the mice treated with the TNF-α depleting antibodies succumbed to infection (Figure 5B). However, 30% of the mice treated with both the IL-1β and the TNF-α depleting antibodies succumbed to CO92*ΔyopH *infection (Figure 5C). These data did not reach significance (p = 0.0675) but suggest that proinflammatory cytokines expressed during CO92*ΔyopH *infection help control this infection.

**Figure 6:**
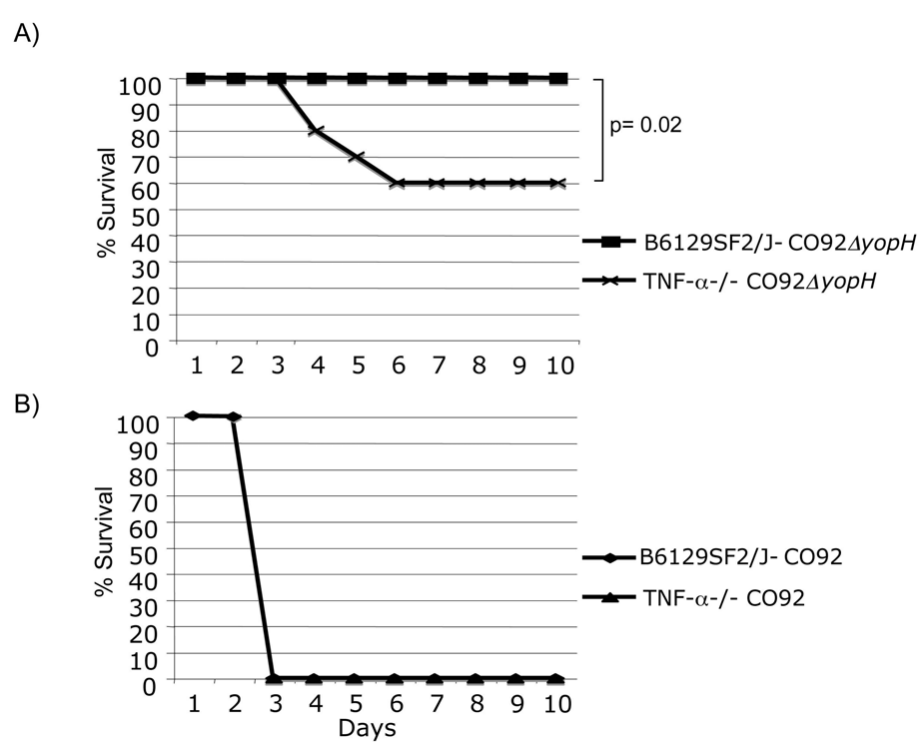
**Mice lacking TNF-α are partially sensitive to CO92Δ*yopH *infection**. B6129F2 control mice or B6;129S-Tnf^tm1Gkl^/J (TNF-α-/-) mice were infected IN with 2 × 10^4 ^CFU of CO92 or CO92Δ*yopH *and then monitored for survival over a ten day period. **A) **TNF-α-/- mice are sensitive to CO92Δ*yopH *infection. **B) **Both TNF-α-/- mice and control mice are sensitive to CO92 infection. Data was analyzed by log-rank analysis and significant comparisons indicated. Data is combined from two independent experiments with 5 mice per group.

Because the site of the infection was the mucosal surface of the nasal cavity and lung, it was possible that systemically administered IgG poorly depletes cytokines at this site. To test our hypothesis in another way, we infected TNF-α deficient mice (TNF-α-/-) and control mice (B6129SF2/J) with CO92*ΔyopH *or CO92. As predicted, the control mice and the TNF-α-/- mice rapidly succumbed to infection with CO92. Consistent with what was observed with out-bred CD1 mice, the B6129SF2/J control mice were completely resistant to infection with 10^7 ^CFU of CO92*ΔyopH *(not shown and Figure 6). However, when the isogenic TNF-α-/- mice were infected with 2 × 10^4 ^CFU CO92*ΔyopH*, significantly (p = 0.0289), 40% of the mice succumbed to infection within 5 days (Figure 6). Altogether, these data suggest that the CO92*ΔyopH *mutant of *Yersinia pestis *is attenuated in multiple strains of wild type mice including CD-1, B6129SF2/J, and C57BL6/j but is partially virulent in mice lacking TNF-α (this study and [4]). These data further suggest that YopH mediated suppression of pulmonary TNF-α is an important virulence promoting mechanism (Figures 3 and 4 as well as data not shown).

### Histopathology of CO92*ΔyopH*infection

Recently, we and others described the development of primary pneumonic plague in the mouse following infection with CO92 [4,5]. The most striking feature of primary plague pneumonia is a 36-hour delay in the inflammatory response to infection, which is characterized by rapid increases in bacterial burden, a lack of inflammatory cytokine and chemokine expression, and scant evidence of inflammation in histopathological examination of lung tissues. CO92*ΔyopH *infection presents a significantly different picture: 1) there is little bacterial replication in the lungs (Fig. 3), 2) CO92*ΔyopH *is severely attenuated in both bubonic and pneumonic plague models (Fig. 1A &1B and [18]), and 3) TNF-α and IL-1β are readily detectable in the BALF of mice infected with CO92*ΔyopH *at 24 hours post-infection (Fig. 4A &4B) suggesting that CO92*ΔyopH *induces a detectable inflammatory response in the lung. Altogether, these data would suggest that the histopathology of CO92*ΔyopH *might be different than that observed during a CO92 infection.

**Figure 7:**
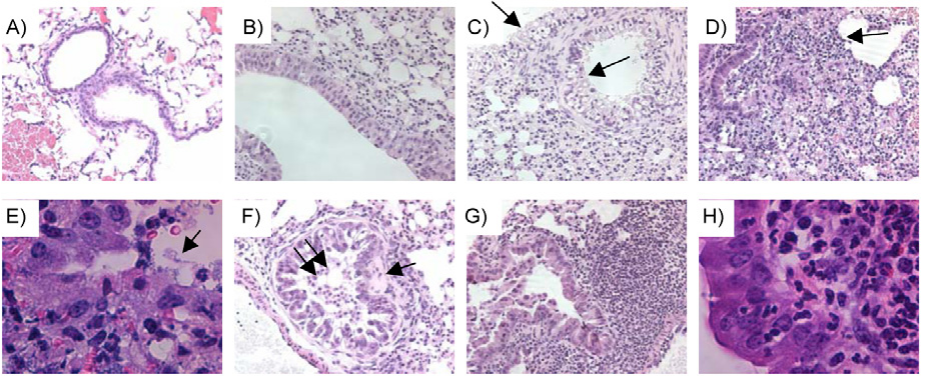
**Histopathology of pulmonary infection with CO92 or CO92Δ*yopH***. CD1 mice were infected IN with 2 × 10^4 ^CFU of CO92 or CO92Δ*yopH *for 24 or 48 hours. Representative 20× images of lungs are presented. **A**) Mouse mock infected for 24 hours with saline. **B**) Mouse infected with CO92 for 24 hours. **C**) Mouse infected with CO92Δ*yopH *for 24 hours with black arrow pointing out vacuolated bronchial epithelium. **D**) Mouse infected with CO92 for 48 hours. Black arrows denote robust neutrophilic inflammation, bacteria, and dead cells. **E**) 100× image of free bacteria in the airways of mice infected with CO92 for 48 hours (black arrow). **F-H**) Mouse infected with CO92Δ*yopH *for 48 hrs. **F**) Vacuolization in conjunction with exudates denoted by the double black arrows and an area of sub-mucosal necrosis denoted by the single black arrow. **G**) Large infiltration of PMNs adjacent to bronchiole. Note that there are no bacteria evident at this time point. **H**) 100× image of infiltration of the bronchial epithelium with PMNs. Data is representative images of 8-10 mice per time point.

To evaluate any differences in histopathology, CD1 mice were infected IN with 2 × 10^4 ^CFU of CO92 or CO92*ΔyopH*. Twenty-four and 48 h post-infection lungs were harvested, fixed, embedded in paraffin, and stained with hematoxylin and eosin. Tissues were examined for inflammatory changes as we have described previously [4,23-25]. Consistent with our previous findings, infection with CO92 for 24 h leads to very subtle changes including congestion (Fig. 7B) and hyperplasia of the bronchial epithelium (Fig. 7B) with little apparent tissue damage. However, infection with CO92*ΔyopH *for 24 h leads to wide spread vacuolization of the bronchial epithelium (Fig. 7C, black arrows), and infiltration of the parenchyma with inflammatory cells. All of the mice infected with CO92*ΔyopH *had extensive vacuolization of the bronchial epithelium as shown in figure 7C. Although there is extensive damage to the bronchial epithelium, the majority of conducting airways remain relatively clear of debris with the exception of the occasional macrophage in the lumen of the airways (Fig. 7C). Consistent with what is seen with the wild-type infection at 24 h, the lower airways and alveolar spaces of the CO92*ΔyopH *infected animals were characterized by congestion (not shown).

At 48 h post infection the histopathology of the CO92*ΔyopH *infection remains very different than that observed during a CO92 infection. The wild-type infection is characterized by large areas of pulmonary consolidation (not shown), a robust inflammatory response composed of mostly of PMNs (Fig. 7D, black arrow), fibrin, exudates in conducting airways (Fig. 7D), and abundant free bacteria (Fig. 7E, black arrow). The inflammatory response to the CO92 infection at 48 h post-infection also includes multiple areas of overt extracellular bacterial growth interspersed with a large number of dead host-cells and cellular debris. These findings were observed in 100% of the mice infected with CO92 for 48 h. CO92*ΔyopH *exhibits several prominent findings at the 48 h time point (Fig. 7F-H). Large pronounced inflammatory lesions composed mostly of PMNs were present in 50% of the mice investigated at this time point (Fig. 7G and 7H). These lesions are different than those observed in the wild-type infection in that bacterial growth is not apparent (Fig. 7G), there is less cell death and cellular debris (compare Fig. 7D and 7G), and there is a pronounced infiltration of the bronchial mucosa with inflammatory cells (Fig. 7H). The other type of lesion common in the CO92*ΔyopH *infected mice is exemplified in Figure 7F and consists of severe vacuolization of the bronchial epithelium. In contrast to what is observed at 24 h (Fig. 7C), necrosis of the bronchial sub-mucosa is apparent in areas adjacent to vacuolated epithelium in 50% of the animals examined (Fig. 7F, black arrow). Unlike the clear airways observed 24 h after CO92*ΔyopH *infection, at 48 h post-infection, there are exudates composed of PMNs, macrophages, and sloughed epithelial cells (Fig. 6F). However, bacteria were not evident in the airways of any of the animals infected with CO92*ΔyopH*. Altogether these data suggest that CO92*ΔyopH *induces a robust inflammatory response prior to what is observed with CO92 infection. Further, CO92*ΔyopH *causes significant vacuolization of the bronchial epithelium that is only occasionally observed during a CO92 infection.

## Discussion

We investigated the effect of YopH deletion on the virulence of *Y. pestis *using a murine model of primary pneumonic plague. YopH, a type-three secretion system effector protein, is a protein tyrosine phosphatase that is involved in inhibiting phagocytosis [14,17], as well as blocking the PI3-kinase pathway [15]. Investigators have studied the effect of YopH deletion using multiple species of *Yersinia*, including *Y. pestis yopH pgm *double mutants utilizing several routes of infection [11,15,17-21]. To date most yopH mutants tested have exhibited decreased virulence and interestingly, lung infections of Balb/c mice with a yopH mutant of the closely related *Yersinia pseudotuberculosis *resulted in a strong attenuation phenotype [19]. However, this has never been tested in a murine model of primary pneumonic plague using WT *Y. pestis *strains.

The attenuation of CO92*ΔyopH *is due to the loss of the protein because virulence can be restored when the CO92*ΔyopH *mutant is complemented with a wild type copy of the YopH gene. Further, it is the actual phosphatase activity of YopH that is required for virulence since a CO92*ΔyopH *mutant complemented with a yopH gene containing an enzymatic inactivating mutation (C403A) is completely attenuated. This is an intriguing finding suggesting that small molecule inhibitors of the YopH phosphatase activity might be useful therapeutics for the treatment of plague.

In addition to evaluating the virulence of CO92*ΔyopH*, we examined the host response to pulmonary infection by testing for differences in pro-inflammatory cytokines expression and lung histopathology. We hypothesized that there could be a host component that contributed to the attenuation of the CO92*ΔyopH*mutant. It has been observed by us and others that infection with *Y. pestis *CO92 leads to a delayed inflammatory response, which results in very high bacterial loads in the lungs, liver and spleen, ultimately leading to the death of the host [4,5,26]. We speculated that alleviation of the anti-inflammatory mechanisms of *Y. pestis *would provide protection for the host.

In the absence of YopH, the host responds to the presence of *Y. pestis *with an early pro-inflammatory response, namely, an increase in TNF-α and IL-1β in the BALF at 24 h post-infection, which diminishes in magnitude by 48 h post-infection. This is different than what is observed with CO92 infection, where an inflammatory response is not observed until 48 h post-infection [4]. In addition, the kinetics of bacterial growth following an intranasal infection with CO92*ΔyopH *is different than the kinetics of CO92 intranasal infection. First, CO92*ΔyopH *is able to persist in the lungs of infected mice, although the bacterial burdens observed are significantly lower than those of CO92 at both at 24 h and 48 h post-infection. It appears that the CO92Δ*yopH *is unable to propagate in the lungs of the host, as demonstrated by the fact that the amount of bacteria detected in the lungs remains approximately the same at 24 h and 48 h post-infection. These data are similar to what is observed with a pulmonary infection with the *Y. pseudotuberculosis yopH *mutant [19]. Additionally, CO92*ΔyopH *is unable to disseminate from the site of infection to either the liver or spleen at 24 h, 48 h, 72 h, or 96 h post-infection. In contrast, infection with wild-type CO92 results in dissemination and high bacterial burdens in the liver and spleen at 48 h post-infection, and the majority of the animals are dead by 72 h post-infection [4]. Viable CO92*ΔyopH *is cleared from the host by 72 h post-infection being undetectable in the lung, liver, or spleen of mice 72 h and 96 h post-infection (data not shown). In light of these data, we hypothesize that the early pro-inflammatory response observed during infection with CO92*ΔyopH *is key in allowing the host to adequately control the bacteria and prevent a fulminate infection.

Additional evidence for this conclusion lies in the differences observed in the histopathology of lungs from mice infected with either wild type CO92 or CO92*ΔyopH *at both 24 h and 48 h post-infection. The lung histopathology of these two infections is strikingly different and provides some insight into the dissimilar courses that these infections follow. At 24 hours post-infection with wild-type *Y. pestis*, there are only subtle changes in the lung tissue with very little tissue damage. Infection with CO92*ΔyopH *at this same time post-infection leads to widespread vacuolization of bronchial epithelium and infiltration of inflammatory cells. It is unclear what leads to the vacuolization, but it is possible that the cyto-toxic effects of YopE and YopO are amplified in the *yopH *mutant [6,8,27]. After 48 hours of infection with CO92, evidence of severe lung pathology is evident including pulmonary consolidation and a heavy infiltration of inflammatory cells comprised predominantly of PMNs. In addition, free bacteria are readily observed throughout the lung tissue. In CO92*ΔyopH *infection, at 48 h post-infection, about 50% of mice exhibit large lesions with PMNs, but strikingly, no bacteria are observed. This provides further evidence that an early inflammatory response to infection with CO92*ΔyopH *might be protective, and contribute to the clearance of *Y. pestis *prior to outgrowth and dissemination of the bacteria.

Other investigators recently observed that when innate immunity and specifically inflammatory immune responses are intact, infection with *Y. pestis *does not cause severe disease. For example, *Y. pestis *modifies its LOS when growing at 37°C, using a tetra-acylated lipid A, which only weakly induces a pro-inflammatory response in the host. At 21-27°C, *Y. pseudotuberculosis *produces a hexa-acylated lipid A, the form of lipid A which is able to elicit potent inflammatory responses from the host [28]. The tetra-acylated lipid A has poor Toll-like receptor 4 (TLR-4) stimulating activity and therefore does not elicit the same robust pro-inflammatory response induced by hexa-acylated lipid A [28]. When Montminy et al genetically modified *Y. pestis *to produce the more potent hexa-acylated lipid A at 37°C, they found that mice did not develop bubonic plague when infected subcutaneously even at doses approaching 10^6 ^mean lethal doses [29]. These data indicate that the TLR-4 response is critical in overcoming infection with *Y. pestis *and that by avoiding this response through LPS modification, *Y. pestis *is able to cause disease in the host [29]. A second study demonstrating the importance of an active inflammatory response early in *Y. pestis *infection showed that when mice were latently infected with gamma-herpes virus there is a significant increase in activated circulating macrophages and the severity of subsequent infection with fully virulent *Y. pestis *was decreased compared to mice which were mock infected with virus [30]. Consistent with the data presented in this study, TLR-4 signaling and activated macrophages are capable of producing and responding to IL-1β and TNF-α to fight against bacterial infection.

YopH is a complex virulence factor that impacts many aspects of the pathogenesis of *Yersinia sp*. Given that YopH is a potent protein tyrosine phosphatase, it is well suited to disrupting host signal transduction pathways. It is interesting to note that the majority of evidence obtained studying the enteropathogenic *Yersinia *suggests that YopH acts to diminish integrin signaling following invasin binding [7,12,17,31,32]. The result of this is that *Y. enterocolitica *and *Y. pseudotuberculosis *are predominantly extracellular pathogens. In contrast, *Y. pestis *CO92 does not express invasin [33], and is a facultative intracellular pathogen but YopH is a required virulence factor suggesting it has additional roles in *Y. pestis *pathogenesis.

We hypothesize that the anti-host activities of YopH are beneficial to the survival of *Y. pestis *within the mammalian host and that YopH contributes to the virulence of the organism by preventing an early pro-inflammatory response at the site of infection; thereby allowing the bacteria to rapidly replicate in the organs of infected mice, overwhelming and killing the host. YopH directly impacts these key aspects of the pathogenesis of plague pneumonia. However, given the number of potential host-pathways that could be targeted by YopH, it is likely that this virulence factor impacts multiple aspects of *Y. pestis *pathogenesis.

## Conclusions

Pathogenic *Yersinia *have evolved numerous mechanisms to survive in their hosts and many of the proteins that support survival in the host are virulence factors. Many of the most potent virulence factors are encoded on the 70 Kb virulence plasmid pCD-1 and several of them have been shown to be critical for causing disease. Amongst the essential plasmid-encoded virulence factors is YopH a protein tyrosine phosphatase that has been shown to be critical for the inhibition of invasion by the enteropathogenic yersiniae. Interestingly, *Y. pestis *is a facultative intracellular pathogen that does not express the Inv protein suggesting that *Y. pestis *YopH plays additional roles in the pathogenesis of this pathogen.

A mutation in *yopH *leads to severe attenuation of virulence in mouse models of primary plague pneumonia. In this study, our data suggests that YopH inhibits the production of IL-1β and TNF-α during the first 24 hours post-infection. The inhibition of IL-1β and TNF-α is a critical step in the pathogenesis of infection because mice deficient in these molecules are partially sensitive to infection with the *yopH *mutant. Further, the YopH phosphatase activity is essential for virulence because mice infected with the CO92Δ*yopH *strain complemented with a wild type copy of the yopH gene succumb to infection with kinetics similar to CO92 infection but complementation with the *yopH-C403A *gene restores complete attenuation. Altogether our data suggests that YopH plays an important role in the subversion of innate immunity during plague pneumonia.

## Methods

### Bacteria

*Yersinia pestis *CO92, a biovar Orientalis strain recently isolated from a case of pneumonic plague, was obtained from the Select Agent Distribution Activity (SADA), Centers for Disease Control and Prevention (CDC), Fort Collins, CO. The strain was confirmed to contain the pigmentation (pgm) locus phenotypically by producing red colonies on Congo red plates and by polymerase chain reaction (PCR). The presence of the low calcium response virulence plasmid (Lcr) was confirmed by PCR of the *lcrV, yopH*, and *yopJ *genes. Virulence in mice was confirmed as described below. The *yopH *deletion mutant was created in *Y. pestis *strain CO92 using the suicide vector pSR47s [34], a derivative of pSR47 [35], as described [18]. The *yopH *mutation was complemented by cloning the CO92 yopH gene and 247bp of 5' UTR into the pCR2.1 plasmid creating pAMC-1. Complemented strains were shown to produce equivalent amounts of YopH after in vitro induction of TTS as described [36]. The YopH-C403A active site mutant was produced on the pAMC-1 plasmid background using the quick-change method as we have previously described creating pAMC-2 [37]. YopH was detected in the TCA precipitated culture supernatant and in whole cell extracts by immunoblot using a rabbit polyclonal anti-YopH antibody kindly provided by James Bliska (SUNY Stony Brook).

### Animal Infections

Six to eight-week old female out-bred CD1 mice (Charles River Laboratories, Willmington, MA) were used for most studies and processed as we have previously described [4,18]. TNF-α deficient mice (B6;129S-Tnf^tm1Gkl^/J stock #003008) and controls (B6129SF2/J stock #101045) were obtained from Jackson Laboratory (Bar Harbor, ME) and used at 6 weeks of age. Fully virulent *Yersinia pestis *strain CO92 or CO92*ΔyopH *was grown for 20 hours at 28°C in Heart Infusion broth (US Biological, Swampscott, MA) supplemented with 0.2% xylose. Cultures were then harvested and washed once with sterile PBS, then diluted in endotoxin-free PBS to approximately 10^6 ^bacteria/mL. Bacterial concentrations were verified by enumerating colony forming units (CFU) on Congo red plates. Mice were anesthetized by intraperitoneal injection of 0.5 mL Avertin (20 mg/mL in PBS, 2-2-2 Tribromoethanol, Sigma, St. Louis, MO) and then intranasally infected with 20 uL of inoculums (10 uL/nare). Intradermal infections were performed by injection of the appropriate dilution of culture in 50 μl volume into the left ear using an insulin syringe as described [38]. Actual CFU's for individual experiments were determined by plating serial dilutions of the inoculums and are reported in the subsequent text and graphics. Mice were euthanized by overdose of isoflurane (Iso-Thesia, Vetus Animal Health, Burns Veterinary Supply, Inc., Westbury, NY) and then cervical dislocation at 24, 48, 72, or 96 hours after infection as indicated. For mice used to determine the virulence of CO92*ΔyopH*, mice were inoculated with up to 2×10^7 ^CFU/mouse, and survival was monitored daily over a period of 10-14 days depending on experimental design. All experiments using *Y. pestis *were performed at Biosafety Level 3, in accordance with approved Institutional Biosafety Committee and Institutional Animal Care and Use Committee protocols.

### Bacterial Burden

The bacterial burden in target organs of mice infected with either *Y. pestis *CO92 or CO92*ΔyopH *was determined as previously described [39]. Briefly, lungs, liver, and spleen were harvested at the indicated times and weighed prior to homogenization. Tissue homogenates were then diluted in PBS and dilutions were plated on Congo red plates. Bacterial growth was evaluated after 72 hours incubation at 28°C. Results are presented as CFU/gram of tissue and representative data from two independent experiments with at least six mice per data point is shown.

### In vivo Cytokine Depletion

IL-1β and TNF-α were depleted by antibody-mediated ablation as we described previously [22]. Briefly, CD1 mice were injected IP with 0.25 mg of rat anti-mTNF-α (clone MP6-XT22, e-Biosciences), hamster anti-mIL-1β (clone B122, e-Biosciences) or a mixture of 0.25 mg of both antibodies one day prior to infection with CO92 or CO92*ΔyopH *and then every third day until the end of the experiment. Control animals received an equivalent concentration of isotype-matched irrelevant rat or hamster IgG respectively. Mice were then monitored daily for survival. Data represents experiments with 10 mice per group.

### Bronchoalveolar lavage

Mice were euthanized and a 1 cm longitudinal incision was made to expose the trachea. Bronchoalveolar lavage (BAL) was performed by catheterizing the trachea using 18 gauge catheters (Beckton, Dickinson Infusion Therapy Systems, Inc., Sandy, UT). Each mouse was lavaged with three 1 mL aliquots of PBS with protease inhibitors (pepstatin, PMSF, aprotinin, leupeptin, Sigma, St. Louis, MO). BAL fluids (BALF) were placed immediately on ice, filtered with a 0.2 μm syringe filter (SFCA, Fisher Scientific, Pittsburg, PA), and stored at -80°C for future analysis. The results are representative of two independent experiments with four to six animals per bacterial strain and time point.

#### ELISA

BALF samples or tissue culture supernatants were diluted as appropriate and used in ELISA assays for mouse TNFα and IL-1β (BD Biosciences Pharmingen, San Diego, CA) following the manufacturers instructions. Eight to twelve samples per data point were used in the assays; each sample was assayed in duplicate.

### Histopathology

Following IN infection, the lungs were harvested at the indicated times and their gross appearance was evaluated at necropsy. At least ten animals per data point were examined. Tissues were fixed in 10 ml of 10% neutral buffered formalin (NBF). Formalin-fixed tissues were then embedded in paraffin and 3-4 μm sections were cut and placed on slides. The tissue sections were then stained with hematoxylin and eosin (H&E). Tissues were examined from at least eight mice per time point and evaluated in blind fashion for inflammatory cell infiltration, bacterial colonization, and presence of inflammatory exudates in airways, edema, necrosis, hemorrhage, and fibrin. Images were captured digitally on a Zeiss Axioscope 2 microscope equipped with a digital camera. Images were processed using the Axiovision V.4 suite of software (Carl Zeiss, Inc., Thornwood, NY).

### Statistical analysis

All results were expressed as the mean ± SEM. Statistical differences were determined using either a two-tailed Student's *t *test or Mann-Whitney non-parametric test as indicated using GraphPad In-Stat3 (GraphPad Software). Survival data was analyzed by log-rank analysis where possible. A value for *p *< 0.05 was considered significant.

## Authors' contributions

Designed experiments, performed experiments, interpreted results and wrote the manuscript: AMC, SSB, and PHD. All authors have read and approve of the final version of this manuscript.
